# Mother to Mother (M2M) Peer Support for Women in Prevention of Mother to Child Transmission (PMTCT) Programmes: A Qualitative Study

**DOI:** 10.1371/journal.pone.0064717

**Published:** 2013-06-05

**Authors:** Amir Shroufi, Emma Mafara, Jean François Saint-Sauveur, Fabian Taziwa, Mari Carmen Viñoles

**Affiliations:** Médecins Sans Frontières, Operational Centre Barcelona-Athens, Belgravia, Harare, Zimbabwe; University of Pittsburgh, United States of America

## Abstract

**Introduction:**

Mother-to-Mother (M2M) or “Mentor Mother” programmes utilise HIV positive mothers to provide support and advice to HIV positive pregnant women and mothers of HIV exposed babies. Médecins Sans Frontières (MSF) supported a Mentor Mother programme in Bulawayo, Zimbabwe from 2009 to 2012; with programme beneficiaries observed to have far higher retention at 6–8 weeks (99% vs 50%, p<0.0005) and to have higher adherence to Prevention of Mother to Child Transmission (PMTCT) guidelines, compared to those not opting in. In this study we explore how the M2M progamme may have contributed to these findings.

**Methods:**

In this qualitative study we used thematic analysis of in-depth interviews (n = 79). This study was conducted in 2 urban districts of Bulawayo, Zimbabwe’s second largest city.

**Results:**

Interviews were completed by 14 mentor mothers, 10 mentor mother family members, 30 beneficiaries (women enrolled both in PMTCT and M2M), 10 beneficiary family members, 5 women enrolled in PMTCT but who had declined to take part in the M2M programme and 10 health care staff members. All beneficiaries and health care staff reported that the programme had improved retention and provided rich information on how this was achieved. Additionally respondents described how the programme had helped bring about beneficial behaviour change.

**Conclusions:**

M2M programmes offer great potential to empower communities affected by HIV to catalyse positive behaviour change. Our results illustrate how M2M involvement may increase retention in PMTCT programmes. Non-disclosure to one’s partner, as well as some cultural practices prevalent in Zimbabwe appear to be major barriers to participation in M2M programmes.

## Introduction

The vast majority of all childhood HIV is acquired vertically [1], [2] with up to 40% of exposed breastfed infants becoming HIV positive [3]. In resource limited settings Prevention of Mother to Child Transmission (PMTCT) programmes can reduce such vertical transmission below 5%; [5]–[9] with the Ministry of Health and Child Welfare (MoHCW) of Zimbabwe aiming to achieve this level of transmission by 2015 [10].

In order to meet this goal Zimbabwe, as elsewhere, must expand access to PMTCT. In 2005 it was estimated that just under half of HIV positive women in Zimbabwe received some form of intervention to reduce vertical transmission of HIV [11].

Expanding PMTCT may be challenging, as programmes are longitudinal and relatively complex, with adoption of recommendations for the extended use of Nevirapine necessitating retention in care throughout the breastfeeding period, which accounts for around a third of transmission [12]. In practice retention is often low [13] and adherence to advice poor [14] due to barriers acting at each stage of the PMTCT cascade.

### Barriers to PMTCT Participation

Busza et al [15] propose a social ecological framework, whereby barriers to successful PMTCT delivery can be divided into individual level factors, peer and family influences, the community context and the social cultural environment.

Individual level barriers include a person’s risk perception as well as their self-efficacy. [16], [17] For those within PMTCT programmes there is some evidence that lack of secondary education may also be associated with poorer adherence to Nevirapine delivery [18].

Peer and family influences have been shown to be particularly important determinants of PMTCT involvement; with lack of partner support reducing the likelihood of a woman engaging with services [19], [20]. Non-disclosure makes it more difficult for women to adhere to PMTCT guidance; as opposed to situations where partners are aware that Nevirapine is being provided to an infant, where adherence is higher [21].

Community context refers to the prevailing attitudes and practices that women perceive to exist among those living in their immediate vicinity. Stigma is an important community level factor that has been shown to affect women’s ability to adhere to HIV treatment [22].

Finally, at the level of the socio-cultural environment, factors such as health and religious beliefs and traditional practices in areas such as infant feeding can run contrary to the recommendations of PMTCT programmes. [23], [24] Gender norms, can also affect the ability of the woman to determine infant feeding practices and may also exacerbate barriers to male involvement in PMTCT [25].

These barriers operate at each level of the PMTCT programme, from testing and diagnosis of the mother, to treatment initiation, testing of the infant, with follow up and re-testing. These steps make up the “PMTCT cascade” and high attrition at each of these steps often results in a “leaky cascade” meaning that the potential benefits of PMTCT may not be realised in practice [26].

Zimbabwe has limited formal health care personnel to address issue such as default from PMTCT programmes, let alone support the wider information and support needs of affected women [27]. Existing semi professional cadres such as village health workers, while often tasked with such responsibilities, face an ever-increasing range of initiatives, limiting the additional time they may spend on PMTCT.

Originally developed in South Africa, Mother-to-Mother (M2M) or “Mentor Mother” programmes employ HIV positive mothers to provide support and advice to HIV positive pregnant women and mothers of HIV exposed babies. These programmes aim to provide psychosocial support and education in areas such as feeding practice as well as promoting retention in care and encouraging disclosure.

Increasingly the benefits of such programmes are being recognised. UNAIDS’ Global Plan for PMTCT [28] highlights the need for community and in particular, female empowerment. While some existing research does suggest that community involvement is important for PMTCT [13], [29] studies of mentor mothers in high income settings show mixed results [30], [31]. Although there is some evidence from a South African plot study of M2M support indicating improved knowledge and retention among PMTCT mothers [32], there is very little evidence at present to demonstrate what benefits established PMTCT programmes have actually been able to deliver.

MSF supported a Mentor Mother programme in Bulawayo, Zimbabwe from 2009 to 2012.

This programme was fully operational in 2 clinic sites (Luveve and Pelandeba) between February 2011 and December 2011. Within this programme it was recommended that all HIV positive pregnant women were asked at antenatal booking or, if failing to attend, at delivery, whether they wished to enrol in the M2M programme.

During the period of February to November 2011 (during which the programme was fully operational in both centres) 535 exposed babies were delivered at MSF supported sites and it was observed that Mothers in the M2M programme were twice as likely to return for testing at 6–8 weeks, compared with mothers who had not enrolled in M2M, 99.2% vs 48.6%, p<0.0005. Among mothers who did return for testing at 6–8 weeks, M2M mothers were more likely to have obtained the result of that test, 99.2% vs 73.3%, p<0.0005) (**see Table S1**).

In this study we have set out to explore using qualitative methods, the perceptions of relevant stakeholders of the M2M programme. This included exploration of interviewees’ views of how M2M involvement may have affected retention in care.

## Methods

Bulawayo is Zimbabwe’s second largest city, the suburbs of Pelandeba and Luveve are approximately 10 and 20 km from the city centre respectively, and are home to clinics providing Antenatal Care (ANC) and Prevention of Mother to Child Transmission (PMTCT) services.

PMTCT services at these sites incorporate routine pre-natal HIV testing and maternal antiretroviral therapy (WHO option A) [33]). HIV exposed infants are tested for HIV at 6–8 weeks old, with mothers of those testing negative provided with Nevirapine on a monthly basis, to reduce transmission of HIV via breast milk.

Mothers enrolled in the Mentor Mother programme receive standard PMTCT care, in addition to a holistic, personalised, package of psycho-social support from a designated mentor mother.

Specific activities under the Mother to Mother mentorship initiative included; health education, information on HIV and STI prevention and care including safer sex practices, birth planning, infant feeding counselling and support based on knowledge of HIV status and HIV adherence counselling,

Mentor mothers coordinated their activities with those of formal healthcare staff through participation in case management meetings, and also supported defaulter tracing throughout the PMTCT period. Finally mentor mothers advised patients from the community or households to visit health care facilities for specialized professional counselling or other psychosocial support services where they deemed this appropriate.

Mentor mothers visited at least four women in a week and spent on average six hours with each woman.

Luveve and Pelandeba are the only sites in Bulawayo providing M2M support.

We conducted in-depth interviews with individuals associated with the M2M programme in Bulawayo, Zimbabwe.

### Study Tools

To begin exploration of the study questions we conducted 3three focus groups, the first formed of mentor mothers, the second of beneficiaries and the third of families of those in the first 2 groups. We used the results to inform the conduct of subsequent in depth interviews. A key informant assisted in planning the focus groups and in developing the interview guide.

### Recruitment and Eligibility Criteria

All beneficiaries included in the study received M2M support at Luveve or Pelandeba health clinics in Bulawayo, Zimbabwe.

We purposively sampled a wide range of individuals associated with the M2M programme in order to compare their responses. This included beneficiaries (mothers enrolled in the M2M programme) as well as mothers who had refused to enter the M2M programme, mentor mothers themselves, family members of those involved with the programme and a range of health care staff providing ante-natal care (ANC) and PMTCT services.

We sought to recruit all mentor mothers who were active during the study period. We requested that all mentor mothers bring with them two beneficiaries to whom they were currently providing support. Family members were recruited by those mothers participating in the study. Women declining to participate in M2M were recruited for inclusion in the study on an on-going basis by clinic health staff, during the study period.

### Interviews

Interviews were conducted in a private area and employed open questioning. Responses were recorded on audiotape or in note form if consent for tape recording was not provided. Interviewees were encouraged to respond in their first language. Interview results were organised according to the main themes that emerged from in depth interviews.

### Analysis of Interview Results

A full anonymous interview transcript was produced in English. The researcher conducting the interviews was a social scientist with no previous M2M involvement.

After completion of fieldwork a stakeholder workshop was held to review interview transcripts and begin identification of key emergent themes. Following this workshop, the interviewer coded all interview transcripts according to the themes identified in the workshop. Additional themes were also identified at this time. The interview transcripts were then reviewed and re-coded by a second researcher.

Finally summaries of coded responses were produced by respondent type, summarising the type and frequency of responses within each theme. These were used to examine patterns in responses within and between respondent types.

### Ethical Issues

The Medical Research Council of Zimbabwe (MRCZ) approved the study. All participants provided written informed consent, in English or a local language. Consent was provided confidentially in a private room to a research assistant not involved with patient care. A standardised consent form conforming to MRCZ and WHO recomemndations was used. No financial incentives were provided, although a meal was provided to compensate individuals for their time. Public transport expenses were also reimbursed. Whether an individual accepted or declined to participate in the study had no impact on their current or future care.

## Results

In depth interviews were completed by 14 mentor mothers, 10 mentor mother family members, 30 beneficiaries (women enrolled both in PMTCT and M2M), 10 beneficiary family members, 5 women enrolled in PMTCT but who had declined to take part in the M2M programme and 10 health care staff working at the sites of interest. Results were first organised thematically and then themes presented according to the level of the social-ecological framework (proposed by Busza et al (15)). This framework was identified as appropriate after thematic organisation of results.

### Individual Level: Improved Self-efficacy and Motivation

Mothers who had been involved with the programme consistently stated that it had given them increased confidence/had been empowering; citing practical beneficial consequences, such as an improved ability to negotiate for family planning.

Beneficiary responses suggest that some of this feeling of empowerment arose through proximity to those managing a similar experience in a positive way (mentor mothers).

Knowledge was felt by beneficiaries to have been empowering, particularly the knowledge that they possessed the power to alter the risk of vertical HIV transmission through adherence to PMTCT protocols.

The quotes below support these points:

“*Knowing that you’re talking to someone who has experienced the same situation has been fulfilling and empowering” (Family member of beneficiary, Pelandeba)*

*“They’re (beneficiaries) being empowered through the programme, they understand the PMTCT programme” (Heath worker, Luveve)*

*“I’ve been empowered to practice safer sex in my home” (Mentor mother, Pelandeba)*

### Peer and Family Level: Addressing Non-disclosure

Lack of disclosure was the main family level barrier to PMTCT involvement, which we identified. Those who had not disclosed to their partner cited uncertainty and fear of the consequences as underlying factors. Beneficiaries reported that Mentor Mothers were able to facilitate disclosure between couples by acting as an intermediary and providing a supportive environment within which women felt confident enough to disclose their HIV status.

Lack of disclosure to one’s partner limited women’s ability both to follow PMTCT protocols and to join the M2M programme, in the latter case because they feared that programme involvement would lead to unintended disclosure of their HIV status.

Although most respondents who had disclosed described beneficial consequences one respondent reported being disadvantaged by having disclosed her HIV status.

The following quotes illustrate women’s fear of disclosure and show that beneficiaries perceived mentor mothers to have been helpful in facilitating disclosure.

*“I am a single mother, my partner disappeared when I told him that I had tested HIV positive during my first ANC visit” (Beneficiary, Luveve)*

*“What I’ve noticed is disclosure to partner and mothers in-law, that’s where the difficulties arrive” (Health worker, Pelandeba)*

*“I decided not to join M2M because I have not disclosed to the people I stay with, I am afraid if they see a frequent visitor who is known in the community they will become suspicious” (Non M2M mother)*

*“I was afraid of disclosing to my husband but my mentor taught me ways of on how to disclose to my husband” (Beneficiary, Luveve)*

### Peer and Family Level: The Role of Men in PMTCT

Many respondents of all types stated that men were disinterested in PMTCT and M2M, although some beneficiaries suggest that this changed after M2M enrolment. Beneficiaries, mentor mothers and husbands of mentor mothers variably attributed low male engagement with the programme to ignorance, fear of being associated with HIV and reluctance to take a woman’s advice.

Many women who had joined the M2M programme cited examples of significant advantageous male behaviour change in areas such as condom use, family planning and HIV testing.

*“As a married woman my husband also got tested… I was also taught how to use condoms and to convince my husband to use condoms, I’m proud of myself now”* (Beneficiary, Pelandeba)

*“It’s only now when I have a mentor mother that my husband is beginning to understand and take interest in the programme”* (Beneficiary, Luveve)

*“Before I had a mentor it was difficult to insist on condom use in my marriage but now it’s okay because the mentor talked to us both, now I can say “no” to my husband”* (Beneficiary, Pelandeba)

### Peer and Family Level: Peer Support

Mentor mothers were generally viewed by beneficiaries as more approachable than formal healthcare staff. They were also viewed by both beneficiaries and some healthcare staff as being better able to communicate with women in the PMTCT programme, principally because their first hand experience was felt to increase their credibility.

*“When they talk to these women they understand better because they also tell them that they’ve been through the programme, it’s not like when a medical person tells them… half the time the women are more attentive to the mentor mother than the nurse” (Health worker, Luveve)*

*“They ask things that they do not understand from mentor mothers because it’s easier for them to ask from one of their own rather than a nurse” (Health worker, Pelandeba)*

*“I’ve observed that mothers usually feel very comfortable talking to these mentor mothers because they’re talking at the same level” (Health worker, Pelandeba)*

One respondent did voice the contrary view that she would prefer to receive her information from someone with formal training and a formal qualification.

### The Community Context: Stigma and Discrimination

Many beneficiaries voiced the opinion that stigma associated with HIV AIDS had reduced in the medium term (5–10 years). Others felt that stigma was still prevalent. Some respondents felt that stigma was, for the most part, targeted towards those with symptomatic disease or visible drug side effects. Some respondents felt that stigma within the extended family was more prevalent that in the community as a whole. Lack of knowledge was cited as one cause of stigma.

None of the mentor mothers interviewed felt they experienced specific stigma as a consequence of their role in the M2M programme.

Some respondents felt that mentor mothers contributed to reducing stigma in the community, through their esteemed position coupled with their openness in respect to their HIV status.

*“Discrimination will never stop. I once experienced it, things like people telling me that my child will die in the end” (Beneficiary, Pelandeba)*

*“Stigma is still there but not so much, I realise people stigmatise those people who are visibly sick” (Beneficiary, Luveve)*

*“My family was never stigmatised because of my participation in M2M, instead we educate our neighbours and other family members about HIV (Mentor mother, Luveve)*

*“The community looks up to them as role models” (Health worker, Pelandeba)*

Although not specific to M2M, clinics were considered by some beneficiaries to be stigmatising and clinic staff recognised that routine practices could lead to stigma as well as unintended disclosure. Further issues identified that relate to the clinic setting are included as **Appendix II**.

### Socio-cultural Environment: Gender Norms

Gender norms in the communities of interest were seen by some beneficiaries to limit their ability to implement PMTCT programme related advice within the household. While we found some evidence of PMTCT programme involvement influencing individual male behaviours we did not find any evidence that this had led to broader socio-cultural changes in the communities of interest. The following quotes illustrate how gender roles may negatively impact PMTCT programme delivery:

*“You must not do things that you are told by your wife” (Family member of mentor, Luveve)*

*“Culturally men do not take advice from a woman” (Beneficiary, Luveve)*

### Socio-cultural Environment: Culture and Community Behavioural Norms

Respondents stated that some traditional practices can prevent adherence to PMTCT protocols, the most commonly cited was “muti” a thin porridge fed to new-borns, which, respondents reported, was believed to ward off evil. Next most commonly reported was the tradition that no one outside a newborn’s family should see a baby in the first 6 weeks of life. Such practices were seen to be enforced by mothers in law, a group perceived to be highly influential in influencing the care of newborns. Other groups seen to promote adherence to traditional practices were the Apostolic sect, faith healers and traditional healers.

In some cases mentor mothers reported success in modifying the way traditional practices were implemented.

*“The baby must be given “muti” within 6 weeks to protect the child from evil” (Family member of beneficiary, Luveve)*

*“Until 6 weeks the baby must not be seen by strangers, making it difficult for the mentor mother to visit” (Family member of beneficiary, Luveve)*

*“I think mostly that Apostolic sect, faith healers, traditional healers, these do not book for ANC services, they believe in home deliveries” (Mentor Mother, Luveve)*

*“(we should involve) especially mothers in-law, because these are the key people who dictate how the baby must be looked after” (Beneficiary, Pelandeba)*

### Retention and Adherence - a Cross-cutting Issue

By addressing multiple barriers to proper health seeking behaviour, as described above, we found evidence that Mentor Mother support had led to improved retention and adherence.

All health care staff interviewed observed that the M2M programme had led to a reduction in patient default as reflected in the following quotes:

*“Our defaulter rate has been reduced because these mothers follow these women up – the ladies are coming up now” (Health worker, Pelandeba)*

*“They managed to follow up on defaulters, as a clinic we did not have the capacity to do that” (Health worker, Luveve)”*

*“Mentors make follow ups and visit mothers at home… Checking my register I’ve seen a decline in defaulter rates” (Health worker, Luveve)*

*“The most important benefit is that I’ve managed to retain (in the PMTCT programme) almost 99% of HIV negative babies” (Health worker, Pelandeba)*

## Discussion

We found that most of the benefits of the M2M programme appear to have been mediated through removing individual as well as peer and family level barriers to appropriate health seeking behaviour.

The main benefits that respondents associated with the M2M programme were empowerment, improved knowledge, improved retention and increased disclosure among participants. These benefits appear to have been facilitated by the mentor’s status as expert patients, which, consistent with other research, enhanced their credibility [34], [35].

Empowerment appears to have facilitated behaviour change in areas such as negotiating for condom use and agreeing family planning with partners.

Women within the M2M programme had a far higher retention at the time of testing their babies for HIV (recommended 6–8 weeks post delivery). This may reflect a positive impact of the PMTCT programme upon retention; alternatively it could be due to favourable prior characteristics of those enrolling in M2M.

In support of the prior hypothesis, clinic staff noted a large overall improvement in retention in PMTCT post M2M and attribute this to M2M. Secondly there is existing research suggesting that community level interventions can have a remarkable effect on retention in AIDS programmes [36], [37]. Thirdly, while retention among non M2M mothers was in line with what has been seen elsewhere in Zimbabwe under routine conditions [38], [39], the very high retention seen among M2M mothers, is higher than any other PMTCT data we have seen either in other MSF programmes or reported elsewhere.

In support of the latter hypothesis we did find that mothers not in the M2M programme were less likely to have disclosed to their partners, which they cited as the principal barrier to involvement.

In summary, women who chose not to enter M2M are likely to be different from those who do, irrespective of the effect of the intervention. Nevertheless the very high absolute level of retention among those enrolled in M2M, and the opinions expressed by respondents, support the assertion that M2M increases PMTCT retention.

### Adapting Programme Delivery in Future

Programmatic data agrees with the health care staff perception that most women offered M2M support will enrol in the programme, yet its ability to assist in achieving national PMTCT goals is limited by lack of scale. Increasing the coverage of such programmes requires a paradigm shift, in settings such as Zimbabwe, where expert patients lack official recognition and hospital centred delivery models dominate the healthcare landscape.

Expansion of M2M programmes could be utilised as a way of decentralising the provision of antiretroviral medication. PMTCT mothers are currently required to attend clinics monthly for Nevirapine resupply. Attendance is poor, in part due to long queues and overcrowded clinics.

It may be beneficial, in future, to explore whether mentor mothers may have a role in distributing antiretroviral (ARV) medication (both for infants and beneficiaries) to mothers, given that the feasibility of utilising non-professionals for ARV distribution has already been demonstrated [40].

Our findings confirm previously documented low male involvement with PMTCT in Zimbabwe [29] and support research which finds that unsupportive male behaviours and perceived gender roles can lead to fear of disclosure among women, thus impairing their ability to seek and to provide effective treatment to both themselves and their child [41]–[43] [30]–[32].

Future research should assess how male involvement can be increased without impairing women’s ability to engage openly with peers in group settings.

### Strengths and Limitations

Respondents, particularly mentor mothers, may have sought to provide a favourable impression of the M2M programme if they felt invested in it. We aimed to address this by also interviewing health care staff. Many of those not wishing to participate in M2M also declined to participate in our study. It is plausible that those who did participate had more favourable health behaviours than those who did not.

Our study was conducted in an urban setting. PMTCT has been noted to be a particular challenge in rural Zimbabwe, with higher attrition than urban PMTCT programmes. [44] In rural districts traditional practices may differ; the distances that mentor mothers would be required to travel to visit beneficiaries would generally increase, limiting generalisability to such settings.

Broadly our findings that M2M offers a wide range of benefit to PMTCT, should be generalisable to Southern African settings and is consistent with existing research [32], [45].

Cultural challenges to PMTCT (such as ‘Muti’ in Zimbabwe) and those responsible for enforcing them are likely to be highly heterogeneous emphasising the need for delivery models to be informed by setting.

Existing research on the impact of M2M upon PMTCT has principally demonstrated improved knowledge among participants (22), with other research also finding that community workers (non M2M) can improve knowledge among M2M mothers (36).

While other research has suggested that community based counselling support/follow up visits can improve retention within HIV/AIDS related programmes (37,39) this is the first study we are aware of within a PMTCT programme with such high levels of patient retention. Our research also highlights wider benefits of Mentor Mother programmes, including improved knowledge of HIV prevention, improved feeding practices, increased disclosure and increased use of family planning among participants.

### Conclusions

Mothers of HIV positive infants have great potential as expert patients and given the relative complexity of PMTCT appear to be much needed. Our results illustrate how M2M involvement may increase retention in PMTCT programmes.

Non-disclosure to one’s partner appears to be a major barrier to participation in M2M.

M2M programmes offer a wide range of potential benefits both to beneficiaries themselves as well as to their children. These include increased disclosure and more favourable health behaviours.

## Supporting Information

Appendix S1 **Results of quantitative analysis of programmatic data.**
(DOC)

Appendix S2 **Clinic level barriers to PMTCT programme delivery.**
(DOC)

Table S1 **PMTCT outcomes at 6–8 weeks for beneficiaries of the mentor mother programme compared to non-beneficiaries.**
(DOC)
