# Supplementary material for: Mother to Mother (M2M) Peer Support for Women in Prevention of Mother to Child Transmission (PMTCT) Programmes: A Qualitative Study
Source: PLoS One. 2013 Jun 5;8(6):e64717. doi: 10.1371/journal.pone.0064717 (PMC3673995; doi:10.1371/journal.pone.0064717)
Supplement: Appendix S1 — Results of quantitative analysis of programmatic data. (DOC) [file pone.0064717.s001.doc]

**Appendix SI: Results of quantitative analysis of programmatic data**

Of 535 exposed babies delivered at the study sites during the period of interest, (February-November 2011) 192 were local residents, and so were due to be followed up at those sites, thus being eligible for inclusion in the M2M programme. We were able to locate the records for 90% (164/192) of these mother-baby pairs, the remainder were not locatable. We removed 4 cases from the analysis, as we could not ascertain whether they had enrolled in the M2M programme.

76% (122/160) of mothers of exposed babies who had delivered during the study period enrolled in the M2M programme. Mothers in the M2M programme were twice as likely to return for testing at 6-8 weeks, compared with mothers who had not enrolled in M2M, 99.2% vs 48.6%, p<0.0005. Among mothers who did return for testing at 6-8 weeks, M2M mothers were more likely to have obtained the result of that test, 99.2% vs 73.3%, p<0.0005) (**table S2**)
