# Supplementary material for: Mother to Mother (M2M) Peer Support for Women in Prevention of Mother to Child Transmission (PMTCT) Programmes: A Qualitative Study
Source: PLoS One. 2013 Jun 5;8(6):e64717. doi: 10.1371/journal.pone.0064717 (PMC3673995; doi:10.1371/journal.pone.0064717)
Supplement: Appendix S2 — Clinic level barriers to PMTCT programme delivery. (DOC) [file pone.0064717.s002.doc]

**Appendix S 2: Clinic level barriers to PMTCT programme delivery**

Clinics were characterised by respondents as being busy, having limited physical space, as well as being female dominated, which may have been off-putting to males. Some beneficiaries felt that they had been dealt with poorly and in an unfriendly manner, by clinic staff. Many beneficiaries described long waiting times at clinics, and in some cases individuals reported having given up trying to obtain Nevirapine due to long time delays.

Some beneficiaries as well as health care staff cited practices in clinics which may lead to stigmatisation or unintended disclosure of an individual’s HIV status, examples of such practices included announcements by clinic staff which unintentionally reveal mothers status. Some respondents appear to have been deterred from attending clinics as a result of the practices and conditions described.

Many beneficiaries cited the financial cost of obtaining family planning as a significant barrier to accessing contraception; complaining that from 6 weeks post delivery, charges for these services were incurred. The following quotes illustrate these points:

***“We wait too long to get baby medication from 7am till around 2pm. At times we’re told to go home and come back the next day. Sometimes we end up stopping giving medication because you have run out.”(beneficiary, Luveve)***

***“The nurses should be sensitive to HIV positive people..........they treat us like a separate group from other patients”(beneficiary, Pelandeba)***

***“I’ve noticed that most of the time the mothers are not free when they’re attending sessions in an open space, they feel their confidentiality is betrayed” (Health worker, Pelandeba)***

***“After 6 weeks from delivery if you go to the clinic (for family planning ) you’re asked to pay $16 consultation fee and then for the chosen method” (beneficiary, Luveve)***

***“In terms of family planning it’s difficult when you do not have money to pay for the consultation fee and the different methods” (Beneficiary, Luveve)***

***“Sometimes as health care staff we stigmatise unknowingly, when we say “Cotri (Cotrimoxazole) mothers this side”(health worker, Luveve)***

***“(they say things like) all code 1 (HIV positive) babies this way” (Beneficiary, Luveve)***
