# Supplementary material for: Mother to Mother (M2M) Peer Support for Women in Prevention of Mother to Child Transmission (PMTCT) Programmes: A Qualitative Study
Source: PLoS One. 2013 Jun 5;8(6):e64717. doi: 10.1371/journal.pone.0064717 (PMC3673995; doi:10.1371/journal.pone.0064717)
Supplement: Table S1 — PMTCT outcomes at 6–8 weeks for beneficiaries of the mentor mother programme compared to non-beneficiaries. (DOC) [file pone.0064717.s003.doc]

Table S1: Selected characteristics as well as outcomes of infant testing at 6-8 weeks for mothers within the M2M programme compared to those not in the M2M programme.

|  | Mothers in M2M | Mothers not in M2M | P value |
| --- | --- | --- | --- |
| n | 38 | 122 |  |
| Median CD4 count | 402 (255 – 585) | 378 (231-3989 | 0.2483 |
| WHO stage III* | 2.8%  (3/108) | 5.9%  (1/17) | 0.499 |
| Baby tested | 99.2%  (121/122) | 48.6%  (17/35) | **<0.0005** |
| Test result positive | 1/ 34 | 0/121 |  |
| Mother notified of test result | 99.2%  (118/119) | 73.3%  (11/15) | **<0.0005** |
|  |  |  |  |

***Percentages calculated for those with available data for each item. **Babies recommended to be tested by DNA PCR 6-8 weeks post delivery. We have included here all babies with a recorded test Result at the time of reviewing programmatic data. (March 2011)**
